# Combinatorial RNA interference as a gene therapy strategy for HIV-1 infection

**DOI:** 10.1186/1742-4690-10-S1-P85

**Published:** 2013-09-19

**Authors:** Francesca Spanevello, Arianna Calistri, Claudia Del Vecchio, Barbara Mantelli, Saverio G Parisi, Giorgio Palù, Marina Cavazzana-Calvo, Cristina Parolin

**Affiliations:** 1 Department of Molecular Medicine, University of Padova, Padova, Italy; 2 INSERM U768 and Descartes University of Paris, Paris, France; 3 Department of Biotherapy Necker Children’s Hospital, Paris, France; 4 Department of Biology, University of Padova, Padova, Italy

## Background

RNA interference (RNAi) is a mechanism of gene-suppression with potential gene therapy applications against chronic viral diseases. Combinatorial RNAi approaches are required to account for viral variability in treating HIV-1 infection, as single short hairpin RNAs (shRNAs) are rapidly rendered ineffective by resistant strains. Although promising, these approaches need to be optimized in terms of target selection, hairpin design and promoter choice in order to provide a highly effective anti-HIV-1 therapeutic strategy.

## Materials and methods

Validated small interfering RNAs (siRNAs) with targets within cellular *CCR5* gene and HIV-1 *tat/rev* and *vif* sequences were inserted into a lentiviral vector under the control of either the U6, the 7SK or the H1 human polymerase III promoters, alone or in different combinations. Alternatively, the siRNAs were simultaneously expressed as an extended shRNA (e-shRNA) under the control of each selected promoter. Silencing efficiencies of the different vectors were compared by means of the luciferase knockdown assay. siRNAs antiviral activity and cytotoxicity were assessed both in cell lines and in human primary cells, including macrophages and CD4+ T lymphocytes.

## Results

Experiments performed on single shRNA-expressing vectors indicated a specific silencing activity of the selected sequences, with the siRNAs targeting the *CCR5* and the *tat/rev* genes leading up to 90% reporter gene knockdown. Activities of the U6 and H1 promoters were similar and superior to that of the 7SK, irrespective of the expressed siRNA. Furthermore, siRNA-CCR5 induced a marked cell surface *CCR5* downregulation in transduced primary macrophages. When different siRNA transcriptional units were combined within a single vector, the silencing activity of each siRNA was not affected by its relative position in transfection experiments. On the other hand, differences were observed in transduced cells challenged with wild type HIV-1. As an alternative combinatorial approach, we designed an extended short hairpin RNA giving rise to three different siRNAs targeting *CCR5*, *tat/rev* and *vif*, under the control of either the U6, the 7SK or the H1 promoter. The obtained results showed that the silencing activity is strictly dependent on the used promoter, with the H1 being the most active, both in terms of target gene knockdown and inhibition of viral replication. In addition, the biosafety of human cell lines and primary cells expressing multiple siRNAs was evaluated and transduction at high multiplicity of infection turned out to affect endogenous microRNA processing.

## Conclusions

This study confirms that combinatorial RNAi is a feasible approach to counteract HIV-1 replication, highlighting some important strengths and pitfalls of different platforms used for multiple siRNAs delivery. The data provide valuable insights for the design and application of reliable combinatorial RNAi that, once shown to be safe and effective *in vivo*, may be next in line for clinical testing.

